# The dopaminergic system mediates the lateral habenula-induced autonomic cardiovascular responses

**DOI:** 10.3389/fphys.2024.1496726

**Published:** 2024-11-21

**Authors:** Yuma Sato, Masayuki Matsumoto, Tadachika Koganezawa

**Affiliations:** ^1^ Department of Neurophysiology, Institute of Medicine, University of Tsukuba, Tsukuba, Ibaraki, Japan; ^2^ Doctoral Program in Medical Sciences, Graduate School of Comprehensive Human Sciences, University of Tsukuba, Tsukuba, Ibaraki, Japan; ^3^ Department of Cognitive and Behavioral Neuroscience, Institute of Medicine, University of Tsukuba, Tsukuba, Ibaraki, Japan; ^4^ Center for the Evolutionary Origins of Human Behavior, Kyoto University, Inuyama, Aichi, Japan

**Keywords:** lateral habenula, cardiovascular, dopamine, ventral tegmental area, stress

## Abstract

The lateral habenula (LHb) has been implicated in stress coping and autonomic control. The LHb regulates the midbrain system of monoamine neurotransmitters such as dopamine, serotonin, and noradrenaline. However, how the LHb regulates autonomic cardiovascular control in stressful situations is unclear. In this study, we examined the participation of the midbrain dopaminergic system in the cardiovascular response elicited by activation of the LHb. We used urethane-anesthetized Wistar male rats. We performed electrical stimulation of the LHb to observe changes in heart rate and blood pressure. Stimulation of the LHb caused bradycardia and a pressor response. Application of a nonselective dopamine receptor antagonist attenuated both the heart rate and the blood pressure changes induced by the LHb. We also tested the effects of blockade of dopamine receptor subtypes in the LHb-induced cardiovascular responses. Application of selective dopamine D_1_/D_5_, D_2_/D_3_, or D_4_ receptor antagonists attenuated the LHb-induced pressor response but did not change the HR response. Furthermore, we examined the effect of inactivation of the ventral tegmental area (VTA) on the cardiovascular response induced by LHb stimulation. Inactivation of the VTA turned bradycardia into tachycardia caused by the LHb stimulation and attenuated the pressor response. Our results indicated that regulation of the dopaminergic system by the LHb mediates the generation of the autonomic cardiovascular response. Dopamine D_1_-like and D_2_-like receptors mediate the sympathoexcitation resulting from the activation of the LHb. The VTA is one of the dopaminergic origins related to the cardiovascular response originating from LHb activation.

## 1 Introduction

The autonomic nervous system, comprising the sympathetic and parasympathetic nervous systems, regulates cardiovascular responses such as changes in heart rate and blood pressure. Stress events cause autonomic cardiovascular changes in animals, which contribute to adaptive behavior and coping with stress. Stress behavior is known as “fight-or-flight” or “freezing” (Kozlowska et al., 2015). Elevation of heart rate and blood pressure is accompanied by “fight-or-flight” behavior evoked by escapable stress (Farah et al., 2004; Hsu et al., 2012), whereas decrease in heart rate and increase in blood pressure are associated with “freezing” provoked by inescapable stress (Yoshimoto et al., 2010; Koba et al., 2016). The cardiovascular center in the medulla modulates these cardiovascular changes. The nucleus ambiguus and rostral ventrolateral medulla are prominent in autonomic heart rate regulation and blood pressure (Stuesse and Fish, 1984; Brown and Guyenet, 1985). The cardiovascular center receives outputs from higher brain structures (Dampney et al., 2002). Since stress-related brain areas also send information to the cardiovascular center, the central command can modulate the cardiovascular center and respond to stressful situations.

The lateral habenula (LHb) is a small brain nucleus located above the thalamus. The neural circuit, including the LHb and its efferent brain areas, could be essential for processing emotion and coping with stress (Matsumoto and Hikosaka, 2007; Velazquez-Hernandez and Sotres-Bayon, 2021). Neurons in the LHb show phasic excitation to aversive events (Matsumoto and Hikosaka, 2007; Matsumoto and Hikosaka, 2009). The possible role of the LHb in regulating autonomic control and stress behavior has also received attention. The electrical or pharmacological activation of LHb neurons causes emotional stress-like autonomic changes, including thermogenesis and cardiovascular control (Ootsuka and Mohammed, 2015). We have reported that electrical stimulation of the LHb in anesthetized rats elicits the cardiovascular response controlled by the sympathetic and parasympathetic nervous systems (Doan et al., 2021). Despite previous evidence that the LHb could modulate both adaptive behavior and autonomic regulation in stress situations, how the LHb regulates autonomic cardiovascular responses induced by stress events is unknown.

One of the essential neurological roles of the LHb is the control of the midbrain monoaminergic system, such as the dopaminergic, serotonergic, and noradrenergic systems (Lecourtier and Kelly, 2007; Bernard and Veh, 2012). The activity of neurons in the LHb regulates dopaminergic neurons in the ventral tegmental area (VTA) and the substantial nigra (Christoph et al., 1986). The VTA received inputs from the LHb via the fasciculus retroflexus (Roman et al., 2020). This midbrain dopaminergic system is the source of the dopaminergic inputs to the prefrontal cortex, striatum, nucleus accumbens, and amygdala (Trutti et al., 2019; Roman et al., 2020). Dopamine activity in the central nervous system has been studied for its function in motor control, learning, and motivation. Previous studies have also shown that dopamine release in the brain and the activity of dopaminergic neurons are altered in aversive situations (Yoshioka et al., 1996; Ilango et al., 2014). Therefore, dopamine signals in stress situations might contribute to behavioral and autonomic responses.

In this study, we aimed to elucidate the participation of the dopaminergic system in the autonomic cardiovascular responses originating from the LHb. Here, we show that electrical stimulation of the LHb decreased heart rate and increased blood pressure. The LHb-induced cardiovascular responses were suppressed by blockade of dopamine receptors. Moreover, inactivation of the VTA attenuated the autonomic cardiovascular responses caused by LHb activation. We propose that the LHb regulates blood circulation via dopamine originating from the VTA.

## 2 Methods

### 2.1 Ethical approval

The animal study was reviewed and approved by the Animal Experimental Committee of the University of Tsukuba (permission numbers: 17-119, 18-027, 19-016, 20-019, 21-030, 22-071, 23-056, and 24-059).

### 2.2 Animal procedures

Wistar male rats (250-380 g, Japan SLC) were used in all the experiments. They were housed under normal laboratory conditions with a 12 h-light/12 h-dark cycle at 25°C and with free access to food and water. At the beginning of each experiment, a rat underwent anesthesia induced with isoflurane (Fujifilm Wako Pure Chemical Corporation) and maintained with urethane solution (250 mg/mL, 1-1.25 g/kg, *i.p.*; Tokyo Chemical Industry Co., Ltd.). After anesthesia was introduced, the rectal temperature was maintained at 36.5°C by use of a thermostatically regulated heating pad. In all the experiments, heart rate (HR) and arterial pressure (AP) were continuously recorded to observe cardiovascular changes. HR was measured by means of electrocardiography recorded with a pair of needle electrodes and amplified by use of a bioelectrical amplifier (AB-651J; Nihon Kohden). AP was recorded from the femoral artery, in which a polyethylene catheter (SP31; Natsume Seisakusho Co., Ltd.) filled with heparinized saline was placed. Mean arterial pressure (MAP) was calculated from the AP. A saline-filled polyethylene catheter (SP31; Natsume Seisakusho Co., Ltd.) was also placed in the femoral vein to administer drugs intravenously.

### 2.3 Electrical stimulation of the LHb of an anesthetized rat

After the surgical procedure, the rat was placed in a stereotaxic apparatus. Burr-hole craniotomy (3 × 4 mm) was performed to stimulate the left LHb. The location of the left LHb (3.6–4.0 mm caudal from the bregma, 0.5–1.0 mm lateral from the midbrain, and 4.4–4.9 mm below the brain cortical surface) was based on a previous report (Doan et al., 2021). The left LHb was stimulated by electrical stimulation (300 µA current intensity, 0.5 m duration, 100 Hz frequency, for 10 s) with a coaxial electrode (coated with polyurethane, 200-µm tip diameter) to record the short-term responses of the HR and AP because LHb neurons show high-frequency (around 100 Hz) and phasic responses to aversive events (Matsumoto and Hikosaka, 2009). We observed the HR and AP response elicited by the electric stimulation, leaving at least 60 s between each stimulation. We confirmed the reproducibility of those cardiovascular responses by several stimulations applied to the LHb, as previously reported (Doan et al., 2021).

### 2.4 *In vivo* pharmacological procedures

We performed intravenous administration of dopamine receptor antagonists to examine the role of the dopaminergic system in cardiovascular responses induced by activation of neurons in the LHb. We used four separate groups of rats treated with different dopamine antagonists: a nonselective dopamine receptor antagonist, clozapine (Fujifilm Wako Pure Chemical Corporation; 1 mg/kg, *i.v*.; n = 5); a D_1_/D_5_ receptor antagonist, SCH23390 (Abcam; 0.2 mg/kg, *i.v*.; n = 5); a D_2_/D_3_ receptor antagonist, sulpiride (Abcam; 10 mg/kg, *i.v*.; n = 5); a selective D_4_ receptor antagonist, L-745870 (0.1 mg/kg, *i.v*.; n = 5). The dose of each antagonist was referenced from previous reports (Mereu et al., 1983; Gioanni et al., 1998; Schwieler et al., 2008; Yan et al., 2012; Ruiz-Salinas et al., 2013; Delcourte et al., 2017). Clozapine, SCH23390, and sulpiride were diluted with saline. L-745870 was diluted with 0.1 M HCl with saline (Fujifilm Wako Pure Chemical Corporation). These drugs were applied from the femoral vein. We first stimulated the LHb in each experiment and observed the HR and AP changes. Secondly, we injected a dopamine receptor blocker into the rat. Then, we observed the effect on the HR and AP changes induced by the electrical stimulation applied to the LHb. The administration of vehicles, *i.e.*, saline or 0.1 M HCl with saline, did not affect the LHb-induced HR and AP changes.

### 2.5 Microinjection procedures

To inactivate the VTA, we made a burr hole (5 × 3 mm) for microinjection into the bilateral brain areas. We injected muscimol, a GABA_A_ receptor agonist (Abcam; 10 mM, 100 nL/side, with 2% Pontamine Skyblue), into 6 rats by use of a microsyringe. The location of the VTA was 5.5 mm caudal from the bregma, 0.6–0.8 mm from the midline, and 8.0 mm below the brain surface. Bilateral injections were performed in 7 min.

### 2.6 Histological confirmation of the brain intervention site

A brain lesion was made by means of an electrical current (direct current, 100 μA, 10 s, once) after a recording to confirm the stimulation site of the LHb. Microinjection sites in the VTA were confirmed by use of Pontamine Skyblue and the microsyringe track with reference to the *Rat brain atlas* (Paxinos and Watson, 1986). The rat was transcardially perfused with 200 mL saline and then with a 10% formalin fixation solution (Fujifilm Wako Pure Chemical Corporation). Next, the brain was obtained and stored in a 10% formalin fixation solution at 4°C for more than 24 h. The brain was cut into 50-µm-thick slices to check the location of the brain lesion and microinjection site. All the sections were immediately scanned by use of a Canoscan 2700F (Canon) or PrimeHisto XE (Pacific Image Electronics).

### 2.7 Data analysis

All the experimental data were digitalized by use of an AD converter (1401plus, Cambridge Electronic Design) and analyzed by use of Spike2 (Cambridge Electronic Design). The digitized data were analyzed by use of Excel 2019 (Microsoft Office) and IBM SPSS Statistics 27 (IBM). All the graphs were also prepared by use of Excel 2019 and Adobe Illustrator. The peak responses elicited by the electrical stimulation applied to the LHb were observed in 6–10 s for the HR response and in 3–6 s for the AP response after the onset of stimulation. Therefore, we calculated the HR, MAP, systolic arterial pressure (SAP), and diastolic arterial pressure (DAP) responses as differences between the mean value for the 10-s prestimulation period and each mean value in each period, i.e., 6–10 s and 3–6 s after the start of the stimulation for HR and for MAP, SAP and DAP, respectively. The response magnitude elicited by the LHb stimulation was expressed as a percent change from the HR and MAP values before the LHb stimulation. All the data were expressed as means ± standard deviations. A paired t-test was used to compare the two groups. Significant *p* values were set at <0.05.

## 3 Results

### 3.1 Involvement of dopamine receptors in the autonomic cardiovascular response induced by LHb activation

**FIGURE 1:**
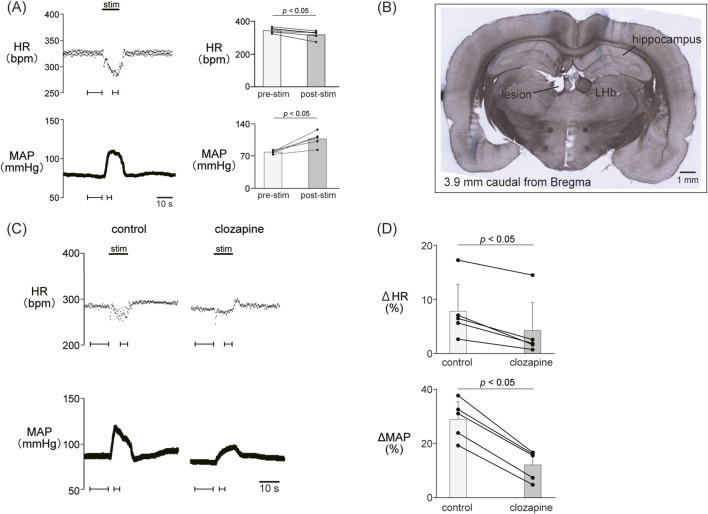
**(A)** Cardiovascular response elicited by electrical stimulation applied to the lateral habenula (LHb). An electrical stimulation for 10 s (top black bar) caused phasic bradycardia and a pressor response. The basal heart rate (HR) and mean arterial pressure (MAP) were stable before and after stimulation to the LHb. The right histograms are the response magnitude of HR and MAP before (pre-stim) and after the LHb stimulations (stim, n = 5). The LHb stimulation decreased HR and increased MAP. **(B)** Image of a brain section for histological confirmation of a stimulation site. The section shows a coronal brain slice 3.9 mm caudal from the bregma. The lesion site of the left LHb was made by a direct current (100 μA, 10 s) after the recording. **(C)** Changes in HR and MAP responses caused by LHb stimulation before and after application of clozapine (1 mg/kg, *i.v.*), a nonselective dopamine receptor antagonist. Black bars at the top of each chart indicate the stimulation periods. **(D)** Change rates of HR (ΔHR) and MAP (ΔMAP) responses to the stimulation of the LHb before (control) and after administration of clozapine (clozapine, n = 5). Administration of clozapine significantly attenuated both the bradycardia and the pressor response caused by LHb activation.

We confirmed the effect of LHb activation on HR and AP. LHb activation significantly decreased the HR (pre-stim, 343.0 ± 14.9 bpm; stim, 318.2 ± 24.2 bpm; *p* = 0.028; n = 5; Figures 1A, B; Table 2) and increased the MAP (pre-stim, 78.8 ± 3.2 mmHg; stim, 107.4 ± 14.4 mmHg; *p* = 0.012; n = 5).

To examine the involvement of the dopaminergic system in the LHb-induced autonomic cardiovascular responses, we observed the LHb-induced cardiovascular responses after intravenous administration of dopamine receptor blockers. The effects of the blockade on baseline HR and AP are shown in Table 1. At first, we systemically administered clozapine, a nonselective dopamine receptor antagonist (1 mg/kg, *i.v.*), and compared the effects of LHb stimulation on HR and AP across the control and clozapine conditions (Figure 1C). The administration of clozapine attenuated both the LHb-induced HR decrease (control, −14.7% ± 5.9%; clozapine, −7.2% ± 4.5%; *p* = 0.002; n = 5; Figure 1D; Table 2) and the MAP increase (control, 28.9% ± 6.5%; clozapine, 12.1% ± 5.0%; *p* < 0.001; n = 5). These results indicated that the dopaminergic system partly mediates LHb-induced bradycardia and MAP elevation.

**TABLE 1:** Baseline heart rate (HR), mean arterial pressure (MAP), systolic arterial pressure (SAP), and diastolic arterial pressure (DAP) before and after application of drugs and injection of muscimol to the ventral tegmental area (VTA) (clozapine, 1 mg/kg, *i.v*., n = 5; SCH23390, 0.2 mg/kg, *i.v*., n = 5; sulpiride, 10 mg/kg, *i.v*., n = 5; L-745870, 0.1 mg/kg, *i.v*., n = 5; muscimol to VTA, 10 mM, 100 nL/side, n = 6).

	HR (bpm)		MAP (mmHg)		SAP (mmHg)		DAP (mmHg)	
	Baseline before drug	Baseline after drug	Baseline before drug	Baseline after drug	Baseline before drug	Baseline after drug	Baseline before drug	Baseline after drug
Clozapine	324.6 ± 28.7	307.4 ± 31.2	90.3 ± 2.6	81.5 ± 10.6	119.6 ± 8.0	108.9 ± 20.2	80.2 ± 4.1	71.7 ± 10.2
SCH23390	339.5 ± 11.0	318.2 ± 7.5*	74.9 ± 1.9	81.2 ± 5.6*	93.7 ± 5.1	101.4 ± 10.1*	67.8 ± 3.1	73.4 ± 4.1
Sulpiride	327.9 ± 26.5	334.0 ± 31.5	78.6 ± 5.8	82.5 ± 2.3	102.1 ± 10.5	107.7 ± 5.8	69.9 ± 5.2	73.2 ± 3.7
L-745870	326.1 ± 22.5	317.2 ± 22.3*	81.9 ± 4.4	85.7 ± 6.0	94.1 ± 6.3	96.3 ± 5.7	79.3 ± 3.3	80.3 ± 6.6
Muscimol to VTA	385.9 ± 33.3	363.4 ± 53.1	74.6 ± 11.6	71.3 ± 8.1	98.3 ± 11.1	93.3 ± 15.1	65.4 ± 11.1	62.4 ± 6.3

Mean value ± SD, *p < 0.05, vs. control, Paired t-test.

**TABLE 2:** Change values of heart rate (HR), mean arterial pressure (MAP), systolic arterial pressure (SAP), and diastolic arterial pressure (DAP) that were induced by the LHb stimulation before (control) and after (drug) application of drugs and injection of muscimol to the ventral tegmental area (VTA) (LHb stimulation, n = 5; clozapine, 1 mg/kg, *i.v*., n = 5; SCH23390, 0.2 mg/kg, *i.v*., n = 5; sulpiride, 10 mg/kg, *i.v*., n = 5; L-745870, 0.1 mg/kg, *i.v*., n = 5; muscimol to VTA, 10 mM, 100 nL/side, n = 6).

	ΔHR (bpm)		ΔMAP (mmHg)		ΔSAP (mmHg)		ΔDAP (mmHg)	
	Control	Drug	Control	Drug	Control	Drug	Control	Drug
LHb stimulation	−25.7 ± 15.3	—	28.7 ± 13.1	—	28.2 ± 13.5	—	26.4 ± 12.9	—
Clozapine	−24.8 ± 14.7	−11.8 ± 13.0*	26.1 ± 6.3	9.5 ± 3.8*	29.0 ± 7.7	11.7 ± 4.7*	24.2 ± 7.5	9.3 ± 4.4*
SCH23390	−16.5 ± 12.5	−17.2 ± 12.2	22.4 ± 4.4	13.3 ± 4.7*	27.0 ± 6.0	14.4 ± 5.2*	21.0 ± 4.4	11.1 ± 4.1*
Sulpiride	−31.8 ± 14.1	−27.1 ± 18.7	23.5 ± 12.0	19.5 ± 14.3	28.1 ± 16.6	22.7 ± 18.6*	19.7 ± 12.7	14.9 ± 15.3
L-745870	−20.8 ± 23.1	−21.2 ± 19.8	23.2 ± 9.6	17.4 ± 10.4*	24.4 ± 11.7	20.6 ± 11.1	20.5 ± 11.2	16.8 ± 10.1
Muscimol to VTA	−15.1 ± 10.9	7.2 ± 17.3*	39.0 ± 5.3	22.5 ± 9.5*	46.8 ± 8.7	27.4 ± 10.5*	38.1 ± 5.1	22.9 ± 8.3*

Mean value ± SD, *p < 0.05, vs. control, Paired t-test.

### 3.2 Involvement of dopamine D_1_-like receptors in the autonomic cardiovascular response induced by LHb activation

**FIGURE 2:**
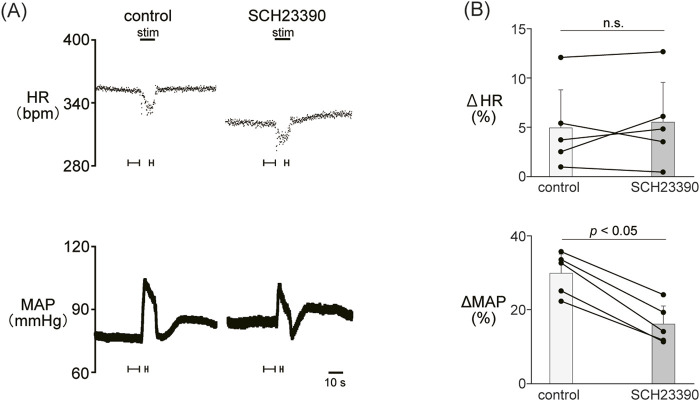
**(A)** Changes in heart rate (HR) and mean arterial pressure (MAP) responses caused by LHb stimulation before and after administration of SCH23390 (0.2 mg/kg, *i.v*.), a dopamine D_1_/D_5_ receptor antagonist. Black bars at the top of each chart indicate the stimulation periods. **(B)** Change rates of HR (ΔHR) and MAP (ΔMAP) responses to the stimulation of the LHb before (control) and after administration of SCH23390 (SCH23390, n = 5). Administration of SCH23390 significantly attenuated the pressor response caused by LHb activation but not the HR response.

Since the blockade of dopamine receptors attenuated the LHb-induced phasic cardiovascular response, we examined the participation of each dopamine receptor subtype in the cardiovascular response. We tested the effect of SCH23390 (0.2 mg/kg, *i.v.*), a D_1_/D_5_ receptor antagonist, on the LHb-induced cardiovascular response. The administration of SCH23390 significantly attenuated the LHb-induced MAP elevation when compared with the control (control, 29.9% ± 5.2%; SCH23390, 16.1% ± 4.9%; *p* < 0.001; n = 5; Figure 2; Table 2). SCH23390 had no significant effect on the HR decrease (control, −4.93% ± 3.9%; SCH23390, -5.51% ± 4.0%; *p* = 0.562; n = 5).

### 3.3 Involvement of dopamine D_2_-like receptors in the autonomic cardiovascular response induced by LHb activation

**FIGURE 3:**
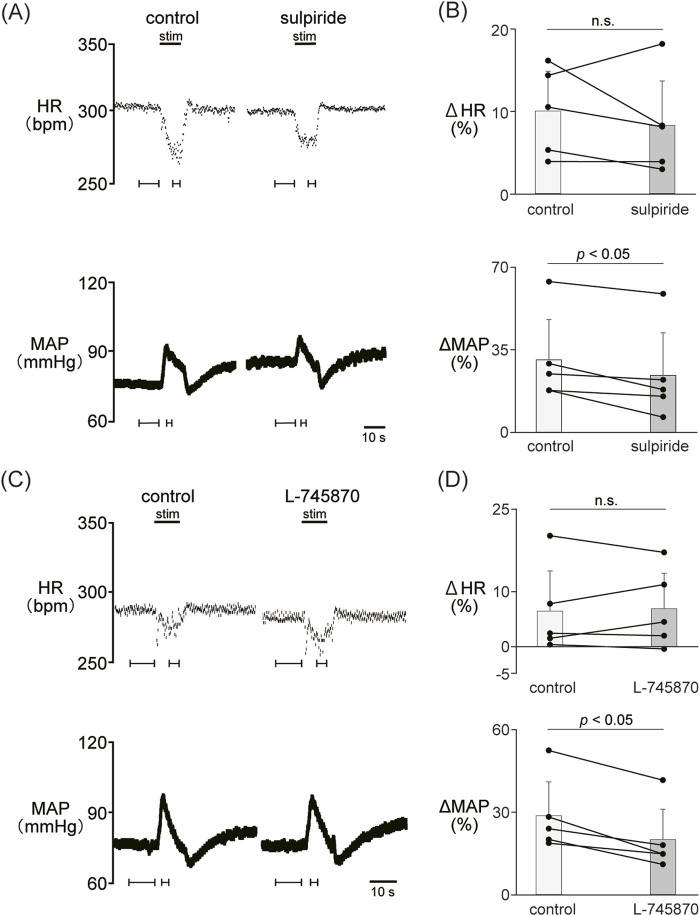
**(A)** Changes in heart rate (HR) and mean arterial pressure (MAP) responses caused by LHb stimulation before and after administration of sulpiride (10 mg/kg, *i.v*.), a dopamine D_2_/D_3_ receptor antagonist. Black bars at the top of each chart indicate the stimulation periods. **(B)** Change rates in HR (ΔHR) and MAP (ΔMAP) responses to the stimulation of the LHb before (control) and after administration of sulpiride (sulpiride, n = 5). Administration of sulpiride significantly attenuated the pressor response caused by LHb stimulation but not the HR response. **(C)** Changes in heart rate (HR) and mean arterial pressure (MAP) responses before and after administration of L-745870 (0.1 mg/kg, *i.v.*), a dopamine D4 receptor antagonist. Black bars at the top of each chart indicate the stimulation periods. **(D)** Change rates of HR (ΔHR) and MAP (ΔMAP) responses to the stimulation of the LHb before (control) and after administration of L-745870 (L-745870, n = 5). Administration of L-745870 significantly attenuated the pressor response caused by LHb activation but not the HR response.

We examined the involvement of the D_2_-like receptor in the cardiovascular response induced by the stimulation of the LHb as well as the D_1_-like receptor. The administration of sulpiride (10 mg/kg, *i.v.*), a dopamine D_2_/D_3_ receptor antagonist, significantly reduced the LHb-induced pressor response (control, 30.6% ± 17.2%; sulpiride, 24.0% ± 18.1%; *p* = 0.029; n = 5; Figures 3A, B; Table 2). On the other hand, the administration of sulpiride did not change the HR response (control, −10.0% ± 4.8%; sulpiride, −8.25% ± 5.4%; *p* = 0.407; n = 5). The administration of a selective D_4_ receptor antagonist, L-745870, significantly attenuated the LHb-induced pressor response (control, 28.6% ± 12.3%; L-745870, 20.0% ± 11.0%; *p* = 0.007; n = 5; Figures 3C, D; Table 2) and did not affect the LHb-induced bradycardia (control, −6.46% ± 7.3%; L-745870, -6.90% ± 6.4%; *p* = 0.736; n = 5).

### 3.4 Involvement of the VTA in the autonomic cardiovascular response induced by LHb activation

**FIGURE 4:**
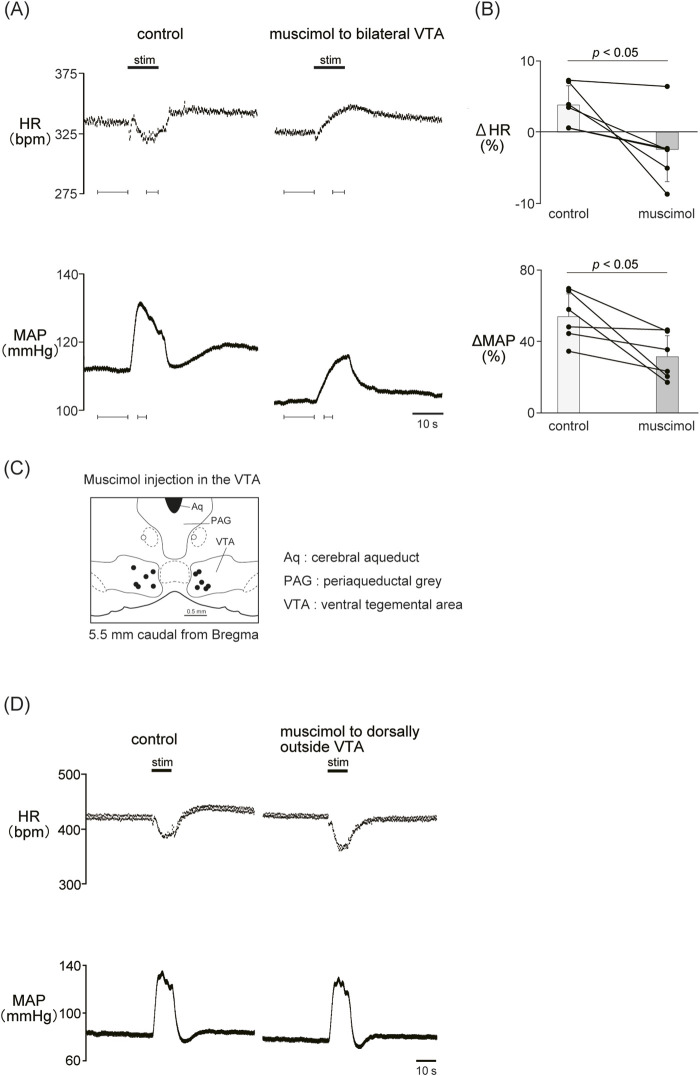
**(A)** Changes in heart rate (HR) and mean arterial pressure (MAP) responses caused by LHb stimulation before and after administration of muscimol (10 mM, 100 nL/side) to the bilateral ventral tegmental area (VTA). Black bars at the top of each chart indicate the stimulation periods. **(B)** Change rates of HR (ΔHR) and MAP (ΔMAP) responses to the stimulation of the LHb before (control) and after microinjections of muscimol to the bilateral VTA (muscimol, n = 6). Inactivation of the bilateral VTA attenuated the MAP response caused by LHb activation. The LHb-induced bradycardia tended to turn into tachycardia after inactivation of the bilateral VTA. **(C)** Injection sites of muscimol in and outside the VTA. The slice is taken from 5.5 mm caudally from the bregma. Black and white dots indicate the injection sites in and outside the VTA, respectively.**(D)** Changes in HR and MAP responses caused by LHb stimulation before and after administration of muscimol (10 mM, 100 nL/side) to the region dorsally outside the VTA. Black bars at the top of each chart indicate the stimulation periods.

Previous results showed that the dopaminergic system is involved in the cardiovascular response induced by LHb activation. The VTA is one of the candidate midbrain dopaminergic areas and relays from the LHb to the limbic system or the medulla. To examine the participation of the midbrain dopaminergic area underlying the cardiovascular response induced by LHb activation, we inactivated the bilateral VTA by locally applying muscimol, a GABA_A_ receptor agonist. The muscimol injections into the bilateral VTA did not change the basal HR and AP (Table 1). The muscimol injections into the bilateral VTA significantly reduced the LHb-induced MAP elevation (control, 53.9% ± 12.7%; muscimol, 31.5% ± 11.8%; *p* = 0.031; n = 6; Figures 4A–C; Table 2). The LHb-induced bradycardia changed to phasic tachycardia (control, −3.81% ± 2.69%; muscimol, 2.41% ± 4.54%; *p* = 0.038; n = 6). On the other hand, administration of muscimol into the region 0.5 mm dorsally outside the VTA did not affect the LHb-induced HR and AP changes (Figure 4D). This result suggested that the VTA mediates the autonomic cardiovascular response originating from LHb activation.

## 4 Discussion

We have here provided evidence that activation of the LHb elicits bradycardia and the pressor response via the dopaminergic system. Blockade of dopaminergic receptors caused changes in the cardiovascular response induced by LHb activation. In the presence of clozapine, a nonselective dopamine receptor antagonist, the LHb-induced bradycardia and pressor response were reduced. Blockade of dopamine D_1_/D_5_, D_2_/D_3_, and D_4_ receptors attenuated the LHb-induced MAP increase. Moreover, inactivation of the bilateral VTA attenuated the LHb-induced bradycardia and pressor response. These results suggest that sympathoexcitation, which leads to a pressor response induced by activation of the LHb, is partly mediated by the dopamine D_1_-and D_2_-like receptors. Especially, the VTA, including dopaminergic neurons, relays the cardiovascular response originating from the activation of the neurons in the LHb.

In this study, administration of a nonselective dopamine receptor antagonist, clozapine, attenuated the LHb-induced bradycardia and pressor response. This attenuation indicated that LHb activation evokes the autonomic cardiovascular response via the dopaminergic system. On the other hand, clozapine also moderately blocks 5-HT_1_, 5-HT_2_, cholinergic, and α-adrenergic receptors (Jann, 2012). Therefore, we also tested the effects of blocking dopamine receptor subtypes on the LHb-induced cardiovascular responses. In the presence of each of the selective dopamine D_1_-and D_2_-like receptor antagonists, the LHb-induced pressor response was also attenuated, but the bradycardia was not affected. This suggested that multiple dopamine subtypes mediate the generation of the pressor response by LHb activation. The possibility remains that blockade of only limited dopamine receptor subtypes cannot reduce the LHb-induced HR decrease because the other dopamine receptor subtypes compensate for the HR changes.

Moreover, inactivation of the VTA also attenuated the LHb-induced bradycardia and pressor response. The LHb neurons project to the midbrain and the brainstem, including the monoaminergic brain regions such as the VTA, rostromedial tegmental nucleus, medial raphe, dorsal raphe, periaqueductal grey, interpeduncular nucleus, and lateral hypothalamus (Hikosaka, 2010; Mizumori and Baker, 2017). We previously reported that the LHb regulates cardiovascular functions partly via the serotonergic system (Doan et al., 2021). Thus, the dopaminergic and serotonergic systems would control the cardiovascular center of the medulla as neural modulators in the neural circuits of the LHb-induced cardiovascular response.

Apart from the brain, dopamine receptors are also expressed in the peripheral vessels, heart, and kidney and are related to humoral regulation and renal function (Bucolo et al., 2019; Qaddumi and Jose, 2021). In our results, administration of dopamine receptor antagonists affected the basal HR and MAP levels. Therefore, blockade of dopamine receptors might affect the humoral system and renal function. However, this study mainly examined the effects of blocking dopamine receptors on the LHb-induced phasic HR and MAP changes. Our previous study also showed that activation of the LHb elicited bradycardia and the pressor response by activation of the cardiac parasympathetic and cardiovascular sympathetic nerves (Doan et al., 2021). Thus, the LHb-induced phasic HR and MAP changes are caused by quick modulation of the autonomic nervous system rather than by the humoral system and renal function. Therefore, the effects of blocking dopamine receptors on the LHb-induced cardiovascular response are central, not peripheral.

The LHb neurons directly and indirectly regulate the VTA dopaminergic neurons. The LHb glutamatergic neurons have strong projections to the rostromedial tegmental nucleus, which innervates the VTA as well as the dorsal raphe (Jhou et al., 2009). The LHb projections to dopaminergic and GABAergic neurons in the VTA are glutamatergic (Omelchenko et al., 2009). The projections from the LHb to the VTA inhibit or activate the VTA dopamine neurons (Matsumoto and Hikosaka, 2007; Brischoux et al., 2009; Jhou et al., 2009; Omelchenko et al., 2009; Yang et al., 2023). Since inactivation of the bilateral VTA attenuated the LHb-induced cardiovascular response in this study, the pathway from the LHb to the VTA, probably dopaminergic neurons, might be involved in the LHb-induced cardiovascular modulation.

A candidate for targets from the LHb-VTA pathway is the amygdala complex. The amygdala complex is roughly divided into three parts: the basolateral nucleus (BLA), the central part (CeA), and the corticomedial nuclei (Sah et al., 2003). The amygdala complex is crucial in cardiovascular regulation and defensive behavior (Soltis et al., 1997; Saha, 2005; Yamanaka et al., 2018; Roelofs and Dayan, 2022). An anatomical study showed that the BLA received dopaminergic inputs from the VTA and substantia nigra (Brinley-Reed and McDonald, 1999). It was also reported that the dopamine D_1_ and D_2_ receptors contribute to the excitability of neurons in the BLA (Kroner et al., 2005). GABAergic neurons in the BLA also project to the CeA neurons (Sah et al., 2003; Roelofs and Dayan, 2022). Our data showed that blockade of VTA activity or D_1_-and D_2_-like receptors attenuated the LHb-induced pressor response. Thus, blockade of dopaminergic inputs to the BLA might cause a change in neural activity between the BLA and the CeA, leading to the pressor response induced by LHb activation. Furthermore, the CeA projections to the medulla control HR and blood pressure (Saha, 2005; Roelofs, 2017; Roelofs and Dayan, 2022). The VTA dopaminergic and nondopaminergic neurons directly innervate the CeA (Avegno et al., 2021). The cardiovascular regulation from the CeA is mediated by the periaqueductal gray, nucleus tractus solitarius, rostroventrolateral medulla, and nucleus ambiguous (Roelofs and Dayan, 2022). It has been known that stress events enhance dopamine release in the CeA and that this modulates inhibitory transmission of CeA neurons via the dopamine D_1_ receptor (Naylor et al., 2010). Under restraint stress, the LHb and the amygdala are activated and show functional connectivity (Durieux et al., 2022). Our data showed that the D_1_/D_5_ receptor blockade attenuated the LHb-induced pressor response. Moreover, inactivation of the VTA attenuated the LHb-induced bradycardia and pressor response. From these findings, at least, the dopaminergic inputs from the VTA to the amygdala may be partly responsible for the LHb-induced HR and blood pressure regulation, although further studies are needed to examine this in detail.

Administration of a dopamine D_4_ receptor blocker, L-745870, reduced the LHb-induced pressor response and did not change the bradycardia. Dopamine D_4_ receptors are expressed in the striatum, which receives dopaminergic projections from the substantia nigra (Rondou et al., 2010). The striatum is related to hypertension (Kirouac and Ganguly, 1993) and autonomic regulation to aversive events (Valenti et al., 2012). Therefore, the substantia nigra-to-the striatum pathway is also likely to participate in the cardiovascular response induced by LHb activation.

In conclusion, this study revealed that regulation from the LHb to the dopaminergic system mediates the generation of the autonomic cardiovascular response. Dopamine D_1_-like and D_2_-like receptors mediate the sympathoexcitation resulting from the activation of the LHb. The VTA is one of the dopaminergic origins related to the cardiovascular response originating from LHb activation. The dopaminergic system originating from the LHb might be essential for stress-related cardiovascular responses.

## Data Availability

The raw data supporting the conclusions of this article will be made available by the authors, without undue reservation.

## Abbreviations

Aq, cerebral aqueduct; BLA, basolateral amygdala; CeA, central part of amygdala; DAP, diastolic arterial pressure; HR, heart rate; i.p., intraperitoneal; i.v., intravenous; LHb, lateral habenula; MAP, mean arterial pressure; PAG, periaqueductal grey; SAP, systolic arterial pressure; Stim, stimulation; VTA, ventral tegmental area.
